# CT-FFR by expanding coronary tree with Newton–Krylov–Schwarz method to solve the governing equations of CFD

**DOI:** 10.1093/ehjimp/qyae106

**Published:** 2024-10-24

**Authors:** Weifeng Guo, Wei He, Yige Lu, Jiasheng Yin, Li Shen, Shan Yang, Hang Jin, Xinhong Wang, Jiang Jun, Xinyang Hu, Jianwen Liang, Wenbin Wei, Jiansheng Wu, Hua Zhang, Hao Zhou, Yanqing Wu, Renqiang Yang, Jinyu Huang, Guoxin Tong, Beibei Gao, Rongliang Chen, Jia Liu, Zhengzheng Yan, Zaiheng Cheng, Jianan Wang, Chenguang Li, Zhifeng Yao, Mengsu Zeng, Junbo Ge

**Affiliations:** Department of Radiology, Zhongshan Hospital, Fudan University, 180 Fenglin Rd, XuHui District, Shanghai 200032, China; Shanghai Institute of Medical Imaging, 180 Fenglin Rd, XuHui District, Shanghai 200032, China; Department of Vascular Surgery, Zhongshan Hospital, Fudan University, Shanghai, China; National Clinical Research Center for Interventional Medicine, 180 Fenglin Rd, XuHui District, Shanghai 200032, China; Department of Vascular Surgery, Zhongshan Hospital, Fudan University, Shanghai, China; National Clinical Research Center for Interventional Medicine, 180 Fenglin Rd, XuHui District, Shanghai 200032, China; National Clinical Research Center for Interventional Medicine, 180 Fenglin Rd, XuHui District, Shanghai 200032, China; Department of Cardiology, Zhongshan Hospital, Fudan University, Shanghai Institute of Cardiovascular Diseases, 180 Fenglin Rd, XuHui District, Shanghai 200032, China; National Clinical Research Center for Interventional Medicine, 180 Fenglin Rd, XuHui District, Shanghai 200032, China; Department of Cardiology, Zhongshan Hospital, Fudan University, Shanghai Institute of Cardiovascular Diseases, 180 Fenglin Rd, XuHui District, Shanghai 200032, China; Department of Radiology, Zhongshan Hospital, Fudan University, 180 Fenglin Rd, XuHui District, Shanghai 200032, China; Shanghai Institute of Medical Imaging, 180 Fenglin Rd, XuHui District, Shanghai 200032, China; Department of Radiology, Zhongshan Hospital, Fudan University, 180 Fenglin Rd, XuHui District, Shanghai 200032, China; Shanghai Institute of Medical Imaging, 180 Fenglin Rd, XuHui District, Shanghai 200032, China; Department of Radiology, The Second Affiliated Hospital, Zhejiang University School of Medicine, Hangzhou 310009, Zhejiang, China; Department of Cardiology, The Second Affiliated Hospital, Zhejiang University School of Medicine, Hangzhou 310009, Zhejiang, China; Department of Cardiology, The Second Affiliated Hospital, Zhejiang University School of Medicine, Hangzhou 310009, Zhejiang, China; Department of Cardiology, The Eighth Affiliated Hospital of Sun Yat-sen University, 3025 Shennan Middle Road, Futian District, Shenzhen 518033, China; Department of Cardiology, The Eighth Affiliated Hospital of Sun Yat-sen University, 3025 Shennan Middle Road, Futian District, Shenzhen 518033, China; Department of Cardiology, The Eighth Affiliated Hospital of Sun Yat-sen University, 3025 Shennan Middle Road, Futian District, Shenzhen 518033, China; Department of Cardiology, The First Affiliated Hospital of Wenzhou Medical University, Nanbaixiang Town, Ouhai District, Wenzhou City, Zhejiang 325088, China; Department of Cardiology, The First Affiliated Hospital of Wenzhou Medical University, Nanbaixiang Town, Ouhai District, Wenzhou City, Zhejiang 325088, China; Department of Cardiology, The Second Affiliated Hospital of Nanchang University, 1 Minde Road, Nanchang City, Jiangxi Province 330006, China; Department of Cardiology, The Second Affiliated Hospital of Nanchang University, 1 Minde Road, Nanchang City, Jiangxi Province 330006, China; Department of Cardiology, Affiliated Hangzhou First People’s Hospital Zhejiang University School of Medicine, No. 261, Huansha Road, Hangzhou 310006, China; Department of Cardiology, Affiliated Hangzhou First People’s Hospital Zhejiang University School of Medicine, No. 261, Huansha Road, Hangzhou 310006, China; Department of Cardiology, Affiliated Hangzhou First People’s Hospital Zhejiang University School of Medicine, No. 261, Huansha Road, Hangzhou 310006, China; Shenzhen Institute of Advanced Technology, Chinese Academy of Sciences, Shenzhen, Guangdong 518055, China; Shenzhen Institute of Advanced Technology, Chinese Academy of Sciences, Shenzhen, Guangdong 518055, China; Shenzhen Institute of Advanced Technology, Chinese Academy of Sciences, Shenzhen, Guangdong 518055, China; Shenzhen Institute of Advanced Technology, Chinese Academy of Sciences, Shenzhen, Guangdong 518055, China; Department of Cardiology, The Second Affiliated Hospital, Zhejiang University School of Medicine, Hangzhou 310009, Zhejiang, China; National Clinical Research Center for Interventional Medicine, 180 Fenglin Rd, XuHui District, Shanghai 200032, China; Department of Cardiology, Zhongshan Hospital, Fudan University, Shanghai Institute of Cardiovascular Diseases, 180 Fenglin Rd, XuHui District, Shanghai 200032, China; National Clinical Research Center for Interventional Medicine, 180 Fenglin Rd, XuHui District, Shanghai 200032, China; Department of Cardiology, Zhongshan Hospital, Fudan University, Shanghai Institute of Cardiovascular Diseases, 180 Fenglin Rd, XuHui District, Shanghai 200032, China; Department of Radiology, Zhongshan Hospital, Fudan University, 180 Fenglin Rd, XuHui District, Shanghai 200032, China; Shanghai Institute of Medical Imaging, 180 Fenglin Rd, XuHui District, Shanghai 200032, China; National Clinical Research Center for Interventional Medicine, 180 Fenglin Rd, XuHui District, Shanghai 200032, China; Department of Cardiology, Zhongshan Hospital, Fudan University, Shanghai Institute of Cardiovascular Diseases, 180 Fenglin Rd, XuHui District, Shanghai 200032, China

**Keywords:** computational fluid dynamics, coronary CT angiography, fractional flow reserve, intermediate lesions, Newton–Krylov–Schwarz algorithm

## Abstract

**Aims:**

A new model of computational fluid dynamics (CFD)-based algorithm for coronary CT angiography (CCTA)-derived fractional flow reserve (FFR) (CT-FFR) analysis by expanding the coronary tree to smaller-diameter lumen (0.8 mm) using Newton–Krylov–Schwarz (NKS) method to solve the three-dimensional time-dependent incompressible Navier–Stokes equations has been developed; however, the diagnostic performance of this new method has not been sufficiently investigated. The aim of this study was to determine the diagnostic performance of a novel CT-FFR technique by expanding the coronary tree in the CFD domain.

**Methods and results:**

Six centres enrolled 338 symptomatic patients with suspected or known coronary artery disease (CAD) who prospectively underwent CCTA and FFR. Stenosis assessment in CCTA and CT-FFR analysis were performed in independent core laboratories. Haemodynamically significant stenosis was defined by a CT-FFR and FFR ≤ 0.80, and anatomically obstructive CAD was defined as a CCTA with stenosis ≥ 50%. Diagnostic performance of CT-FFR was evaluated against invasive FFR using receiver operating characteristic (ROC) curve analysis. The correlation between CT-FFR and invasive FFR was analysed using the Spearman correlation coefficient and Bland–Altman analysis. Intra-observer and inter-observer agreements were evaluated utilizing the intraclass correlation coefficient (ICC). In this study, 338 patients with 422 targeted vessels were investigated, revealing haemodynamically significant stenosis in 31.1% (105/338) of patients and anatomically obstructive stenosis in 54.1% of patients. On a per-vessel basis, the area under the ROC curve for CT-FFR was 0.94 vs. 0.76 for CCTA (*P* < 0.001). Per-vessel accuracy, sensitivity, specificity, positive predictive value, and negative predictive value were 89.8%, 89.3%, 90.0%, 79.0%, and 99.2%, respectively, for CT-FFR and were 68.4%, 82.8%, 62.3%, 48.1%, and 89.6%, respectively, for CCTA stenosis. CT-FFR and FFR were well correlated (*r* = 0.775, *P* < 0.001) with a Bland–Altman bias of 0.0011, and limits of agreement from −0.1509 to 0.1531 (*P* = 0.770). The ICCs with CT-FFR for intro- and inter-observer agreements were 0.919 (95% CI: 0.866–0.952) and 0.909 (95% CI: 0.851–0.945), respectively. The average computation time for CT-FFR analysis was maintained at 11.7 min.

**Conclusion:**

This novel CT-FFR model with the inclusion of smaller lumen provides high diagnostic accuracy in detecting haemodynamically significant CAD. Furthermore, the integration of the NKS method ensures that the computation time remains within an acceptable range for potential clinical applications in the future.

## Graphical Abstract

**Graphical Abstract:**
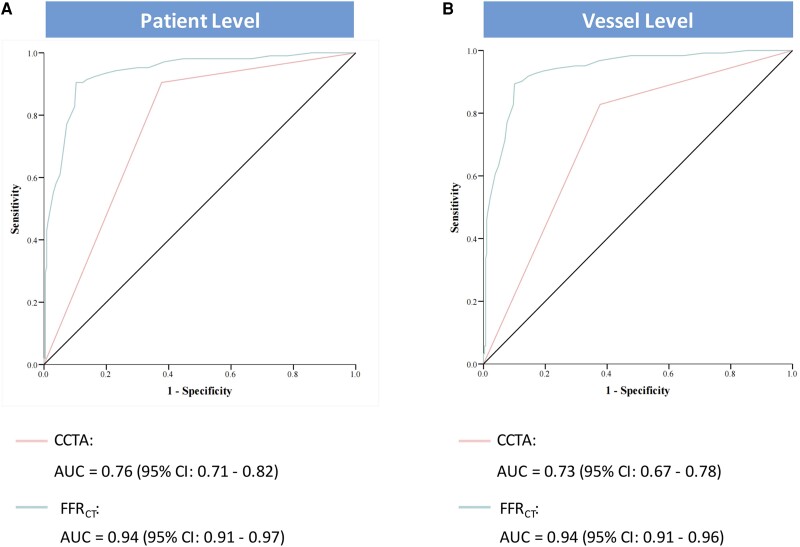
Diagnostic performance of CCTA and CT-FFR to identify haemodynamically significant coronary artery disease. The receiver operating characteristic curve and corresponding area under the curve describing the diagnostic performance of CCTA and CT-FFR to identify haemodynamically significant coronary artery disease as defined by invasive coronary angiography with fractional flow reserve at (*A*) the patient level and (*B*) the vessel level.

## Introduction

Coronary computed tomography angiography (CCTA) has become emerged as a reliable non-invasive diagnostic tool to identify the presence or absence of coronary lumen stenosis.^1,2^ However, CCTA often reveals intermediate coronary artery stenosis, leading to the need for further functional testing.^3^ Computational fluid dynamics (CFD)-based or machine learning (ML)-based techniques have been utilized to simulate coronary CT angiography-derived fractional flow reserve (CT-FFR) of the reconstructed coronary tree, allowing for the assessment of lesion-specific ischaemia using a single resting CCTA.^4–10^ Recent meta-analysis studies have reported a per-vessel sensitivity of 0.85 and a per-vessel specificity of 0.82, showing moderate diagnostic performance. However, there is considerable variation in specificity across different studies compared with sensitivity.^11^ The current CT-FFR simulation models primarily focus on larger lumen diameters (>1.5 mm) of the coronary tree. This limitation may impact the accuracy of CT-FFR since downstream flow in the vascular bed is influenced by vessel diameters in the CFD model.

This paper studies a novel CFD-based algorithm for CT-FFR analysis, which extends the analysis to include smaller-diameter lumen in the reconstructed three-dimensional (3D) coronary artery from CCTA images. In CFD, the effects of blood flow in the downstream arterioles and capillaries are coupled through outlet boundary conditions. Therefore, extending the reconstructed coronary tree to smaller-diameter lumen provides a more realistic model and improves the accuracy of CT-FFR estimation. However, the inclusion of this smaller-diameter lumen presents a computational challenge to the CFD algorithm. To address this challenge, a parallel scalable and robust Newton–Krylov–Schwarz (NKS) method was employed to effectively solve the flow equations and derive the CT-FFR calculations (Artery Technology, Inc., Hangzhou, China).^12,13^ The validation of this method has been demonstrated that the sensitivity of CT-FFR was improved when extending the reconstructed coronary tree to a smaller lumen diameter (0.8 mm) and use the NKS method to solve the flow equations.^14^ However, the study’s retrospective nature and the small sample size limit the generalizability of the findings.

The aim of the present study was to further investigate the diagnostic performance of CT-FFR by extending the reconstructed coronary tree to smaller-diameter lumen and compare CT-FFR results with the invasive FFR, which served as the reference standard.

## Methods

### Study population

This is a prospective, multicentre, noninterventional observational study that took place in six medical centres in China. The primary objective of the study was to assess the performance of CT-FFR, which was simulated by expanding the coronary tree in CFD models. The comparison was made with CCTA to evaluate lesion-specific ischaemia in individuals suspected of having coronary artery disease (CAD). Invasive FFR measurements were used as the reference standard for validation. It is important to note that this study complies with the Declaration of Helsinki and the study protocol received approval from the local institutional review board at each of the six participating centres. Furthermore, all study subjects provided written informed consent prior to their participation in the study.

In this study, symptomatic patients with suspected or known CAD who were referred for clinically indicated invasive coronary angiography (ICA) were prospectively recruited. Patients were subsequently subjected to research-indicated CCTA, ICA, and FFR measurements. To ensure consistency and reliability of the results, certain exclusion criteria were applied. These included patients with previous coronary intervention or coronary bypass surgery; contraindications to beta-blocking agents, nitroglycerine, or adenosine; suspected acute coronary syndrome; myocardial infarction within the last 30 days prior to CCTA or between CCTA and ICA; and a body mass index exceeding 40 kg/m^2^. Consequently, the study specifically included subjects who had between 30% and 90% stenosis of ≥1 major epicardial vessels with a minimum diameter of 2 mm by CCTA, and whose invasive FFR measured in that vessel within 30 days of CCTA.

### CCTA acquisition and analysis

CCTA was performed by using single- or dual-source CT scanners with four CT scanners: a 320-row detector CT scanner (Aquilion One Vision; Canon Medical Systems Corporation), a 128-section multidetector CT scanner (Definition AS; Siemens Healthineers), a third-generation dual-source CT scanner (SOMATOM Force; Siemens Healthineers), and a 256-section wide-detector CT scanner (Revolution HD; GE Healthcare). Prior to the scanning protocol, sublingual nitroglycerine was administered to all patients and metoprolol only if necessary, aiming for a heart rate ≤ 65 beats/min in accordance with Society of Cardiovascular Computed Tomography guidelines.^15^ An initial nonenhanced scan for calcium scoring was performed using prospectively ECG triggered scan with tube voltage of 120 kV. CCTA was performed using prospective electrocardiographic triggering in all patients. The scan was triggered using an automatic bolus-tracking technique with a region of interest placed in the descending thoracic aorta. In the event of a heart rate ≤ 65 beats/min or >65 beats/min, the recommended R–R scan intervals were 65–75% and 40–70%, respectively. Data acquisition was performed with 100 or 120 kV tube voltage in patients weighing ≤70 or >70 kg.

For the image reconstruction, an iterative reconstruction technique was employed. The CCTA images were sent to a central CT-FFR core laboratory (Artery Technology, Inc., Hangzhou, China), and expert evaluators independently assessed the images for various artefacts such as motion, noise, contrast, and blooming. This evaluation was crucial in selecting appropriated cases for CT-FFR analysis. CCTA datasets were evaluated using axial images and multiplanar reconstructions by local observers with extensive experience in cardiac imaging. Vessel segments with a diameter of 2 mm or more were evaluated for lumen narrowing and all vessels of interest were classified according to societal guidelines.^16^ The classifications included: normal (0% stenosis), minimal (<25% stenosis), mild (25–49% stenosis), moderate (50–69% stenosis), severe (70–99%), and occluded (100% stenosis). Any stenosis deemed ≥50% was considered anatomically significant.

### CT-FFR analysis

Patient-specific CT-FFR calculations were conducted at the Artery Technology, Inc. core laboratory using a dedicated algorithm that is based on the NKS method.^14^ All computations were conducted on the Tianhe 2A supercomputer located at the National Supercomputer Center in Guangzhou, China. The CT-FFR analysis was performed in a blinded manner, independent of the results obtained from the invasive FFR measurements. More details regarding CT-FFR analysis based on the NKS method were provided in Supplementary data online, *File S1*.

To assess the inter-observer agreement and variability between different operators in the CT-FFR calculation, an additional operator independently performed the post-processing for 50 randomly selected cases. In addition, this second operator repeated the post-processing for these cases approximately one month after the first analysis to evaluate intra-observer agreement and variability. For each case and vessel, CT-FFR was computed at proximal, middle, and distal segments by each operator. To ensure consistency in the CT-FFR measurements across different operators, anatomic landmarks such as calcium deposits and/or side branches were used as reference points. These landmarks guarantee the CT-FFR calculations to be performed at the same location by different operators. Functionally significant CAD was defined as CT-FFR ≤ 0.80.

### ICA and FFR

ICA was performed by skilled interventional cardiologists following local standards. During ICA, FFR measurements were obtained in at least one vessel with a diameter of ≥2 mm and a stenosis of ≥30%. The selection of the vessel for FFR measurement was at the discretion of the operator, who was blinded to the CT findings. To measure FFR, an FFR pressure wire (St Jude Medical, Minneapolis, MN) was positioned distal to the stenosis of interest and hyperaemia was induced by continuous administration of intravenous adenosine (140 mg/kg/min). Consistent with previous studies,^17^ segments showing angiographic total or subtotal occlusion were assigned an FFR value of 0.50. Lesion-specific ischaemia was defined as a FFR value of ≤0.80, while FFR values between 0.75 and 0.85 were considered as the ‘grey zone’. To ensure accurate co-registration of the location of the invasive FFR measurement and CT-FFR, an independent observer without knowledge of angiographic or functional results, identified the location of the invasive FFR sample on fluoroscopy images. This observer then registered the corresponding location as an anatomical landmark on the CCTA images, allowing for precise alignment and comparison between the two techniques.

### Sample size calculation and statistical analyses

The coprimary endpoints of this study were to evaluate the sensitivity and specificity of CT-FFR using invasive FFR as the reference standard with a clinical significance cut-off value of 0.80. The null hypothesis for sensitivity and specificity was set at 80% and 65%, respectively. The assumed performance goals for sensitivity and specificity to estimate the sample size were based on the NXT trail,^17^ which were 86% and 79%, respectively. Because of the relatively low incidence of ‘positive’ subjects, the sample size was driven by the sensitivity. Considering the ratio of positive to negative lesions of 1:3, type I error (α) = 0.05 (two-sided), statistical power (1-β) of 80% and projected 20% data loss, a minimum of 312 patients was required for the study. In addition to the coprimary endpoints, secondary endpoints included assessing the inter- and intra-observer agreements of CT-FFR, its correlation with FFR, its accuracy, positive predictive value (PPV), negative predictive value (NPV), and receiver operated characteristics area under the curve (AUC) compared with invasive FFR. These secondary endpoints aimed to provide further insights into the performance and reliability of CT-FFR in diagnosing CAD.

Continuous variables were reported as mean ± standard deviation (SD) or median with interquartile range, and compared using the Student’s *t*-test and Mann–Whitney *U* test. Categorical variables were presented as counts and percentages. The Spearman correlation coefficient and Bland–Altman analysis were used to analyse the correlation between CT-FFR with invasive FFR. Intra-observer and inter-observer agreements were assessed using the concordance correlation coefficient and variability in assessment of CT-FFR were determined using Bland–Altman analysis. For AUC comparisons in patients with stenoses 30–90%, analyses were performed on both the per-patient and per-vessel levels, following the method by DeLong *et al*.^18^ Diagnostic performance was obtained by calculating the accuracy, sensitivity, specificity, PPV, and NPV with two-sided 95% confidence intervals (CIs) using the Clopper–Pearson exact method. A significance level of *P* < 0.05 was considered statistically significant. One vessel had missing CT-FFR, and two had missing invasive FFR data. Missing data were handled by exclusion of these vessels. Because the results based on both of these methods did not differ materially, we present the analyses excluding the vessel with missing CT-FFR or FFR. Statistical analysis was carried out using SPSS, version 26.0 (Armonk, NY: IBM Corp) and MedCalc, version 20 (MedCalc Software Ltd, Ostend, Belgium).

## Results

### Patient and lesion baseline characteristics

**Figure 1:**
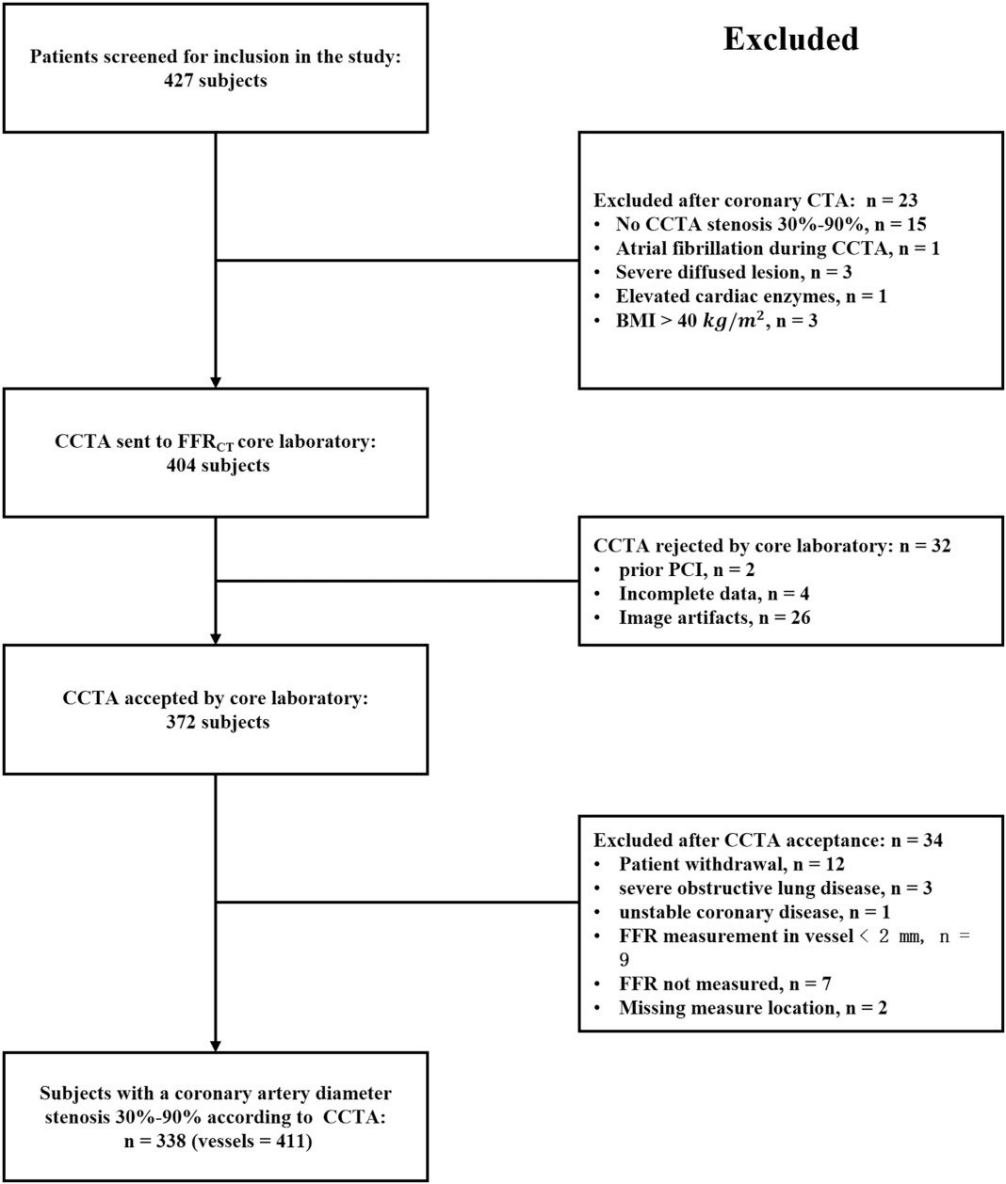
Study enrolment. CCTA, coronary CT angiography; FFR, fractional flow reserve; CT-FFR, coronary CT angiography-derived fractional flow reserve; BMI, body mass index; PCI, percutaneous coronary intervention.

A total of 360 patients were screened for inclusion in this study between July 2020 and May 2022 (*Figure 1*). Nine patients (2.5%) were excluded immediately after CCTA due to specific criteria, while 5 (1.4%) patients were rejected by the core laboratory due to inadequate image quality or incomplete data. Additionally, four patients (2.2%) were excluded due to issues with the FFR measurement, and four patients (2.2%) were excluded from the primary endpoint analysis due to the absence of 30–90% stenosis by CCTA. The reasons for exclusion are detailed in *Figure 1*. Consequently, a total of 338 subjects formed the basis for the primary endpoint analysis. Comparison of FFR and CT-FFR was performed in 411 vessels. The baseline characteristics of the patients are presented in *Table 1*, which includes a mean Agatston score of 236. The average time interval between CCTA and ICA was 13 days. The per-patient and per-vessel characteristics of CCTA, ICA, CT-FFR, and FFR are presented in *Table 2*.

**Table 1:** Patient baseline characteristics (*n* = 338)

Age, year	63 ± 9
Gender	
Male	65.4 (221)
Female	34.6 (117)
Body mass index, kg/m^2^	24.74 ± 3.12
Risk factors	
Hypertension	61.5 (208)
Hypercholesterolaemia	27.2 (92)
Diabetes mellitus	22.5 (76)
Smoking	26.9 (91)
Symptoms	
Typical angina	52.7 (178)
Atypical angina	14.2 (48)
Nonanginal chest pain	12.4 (42)
Dyspnoea	46.7 (158)
Medical therapy	
Platelet inhibitor	16.3 (55)
Statin	50.9 (172)
β-Blocker	18.0 (61)
ACE inhibitor/ARB	32.0 (108)
CCB	30.8 (104)
Anticoagulant	41.1 (139)
Diuretic	10.9 (37)
Peroral antidiabetic	18.6 (63)
Insulin	7.1 (24)
Antianginal agent	15.7 (53)
LVEF, %	65.9 ± 6.8

Values are mean ± SD, or *n* (%).

ACE, angiotensin-converting enzyme; ARB, angiotensin receptor blocker; CCB, calcium channel blocker.

**Table 2:** Patient and vessel characteristics according to CCTA, ICA, CT-FFR, and FFR

Vessel	
LAD	65.9 (271)
LCX	12.7 (52)
RCA	20.9 (86)
LM	0.5 (2)
Patients with CCTA diameter stenosis	
Diameter stenosis < 50%	45.9 (155)
Diameter stenosis ≥ 50%	54.1 (183)
Vessels with ICA diameter stenosis	
Diameter stenosis < 50%	48.9 (201)
Diameter stenosis ≥ 50%	51.1 (210)
Reference diameter, mm	3.5 ± 0.8
Agatston score	162.1 ± 236.2
Patients with FFR	0.84 ± 0.10
≤0.8	31.1 (105)
>0.8	64.8 (219)
Patients with CT-FFR	0.84 ± 0.10
≤0.8	35.2 (119)
>0.8	64.8 (219)
Vessels with FFR	0.84 ± 0.10
≤0.8	29.7 (122)
>0.8	70.3 (289)
Vessels with CT-FFR	0.84 ± 0.12
≤0.8	33.6 (138)
>0.8	66.4 (273)
Patients with FFR ≤ 0.8 in >1 vessel	13.3 (45)

Values are mean ± SD, or *n* (%). *N* = 338 patients and 411 for vessels.

CCTA, coronary CT angiography; ICA, invasive coronary angiography; FFR, fractional flow reserve; CT-FFR, coronary CT angiography-derived fractional flow reserve; LAD, left anterior descending artery; LCX, left circumflex artery; RCA, right coronary artery; LM, left main.

### Correlation of CT-FFR to FFR, reproducibility, and operating time of CT-FFR

**Figure 2:**
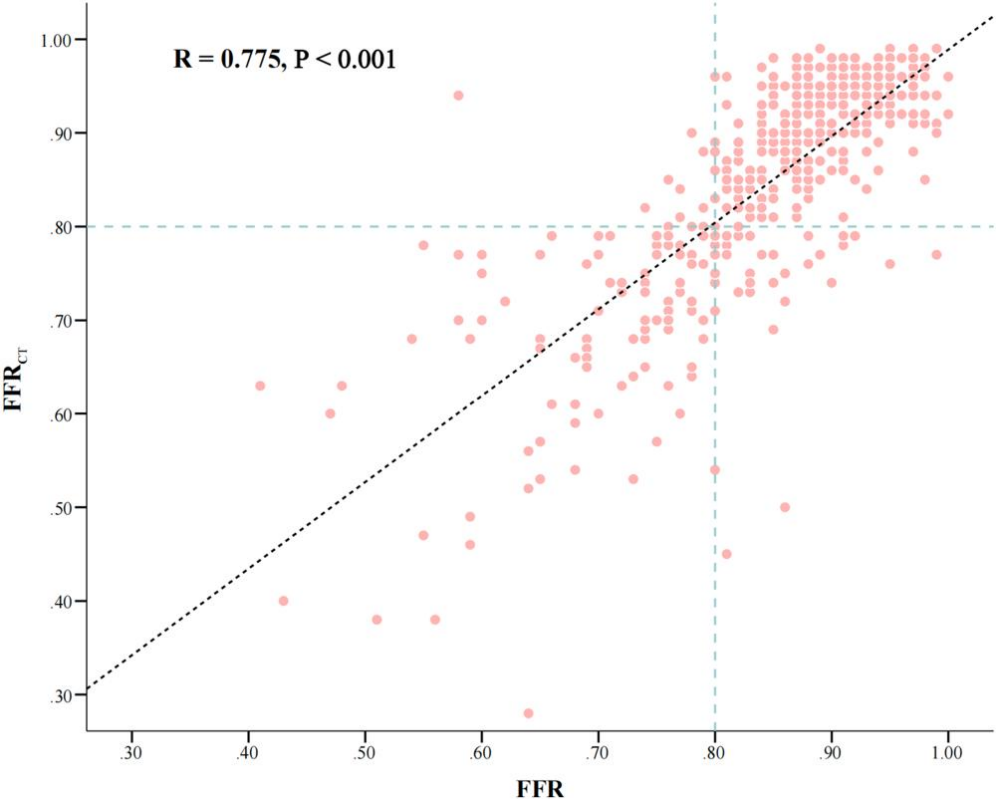
Correlation of CT-FFR to FFR. A good correlation (*r* = 0.78) is observed. Abbreviations as in *Figure 1*.

**Figure 3:**
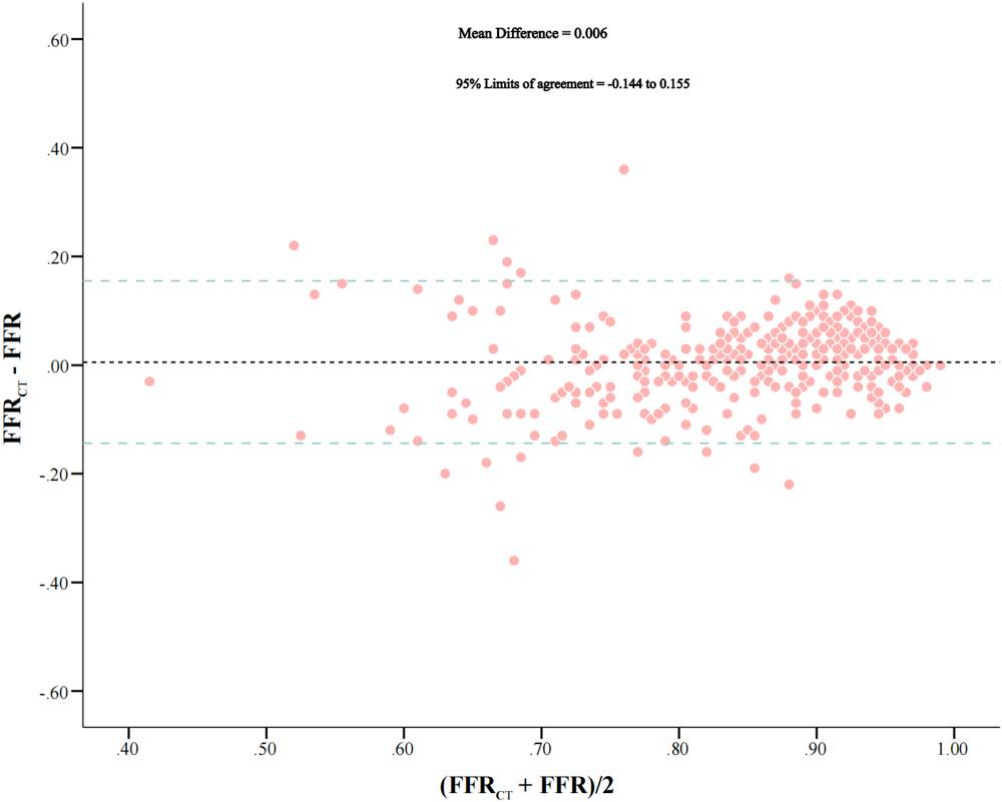
Bland–Altman plot of FFR and CT-FFR on a per-vessel basis. A slight systematic overestimation of computation of fractional flow reserve from CT-FFR as compared with FFR is observed. Abbreviations as in *Figure 1*.

Overall, CT-FFR was highly correlated with FFR (CT-FFR = 0.924 ∗ FFR + 0.065, *r* = 0.775, 95% CI: 0.720–0.824) (*Figure 2*). Bland–Altman plot analysis showed no systematic differences between CT-FFR and FFR, with slight overestimation of CT-FFR compared with FFR of 0.006 [95% limits of agreement (LoA): −0.144–0.155, *P* = 0.770] (*Figure 3*).

The average time per patient referring to the computation of flow simulation required for CT-FFR analysis was 11.7 ± 2.6 min. The concordance correlation coefficients with CT-FFR for intro- and inter-observer agreements were 0.919 (95% CI: 0.866–0.952) and 0.909 (95% CI: 0.851–0.945), respectively. There was a mean intra-observer variability of 0.018 ± 0.058, and the 95% LoA were −0.096–0.132. The mean inter-observer variability was 0.049 ± 0.062 (95% LoA: −0.072–0.170).

### Diagnostic performance of CT-FFR vs. CCTA for diagnosis of ischaemia

**Figure 4:**
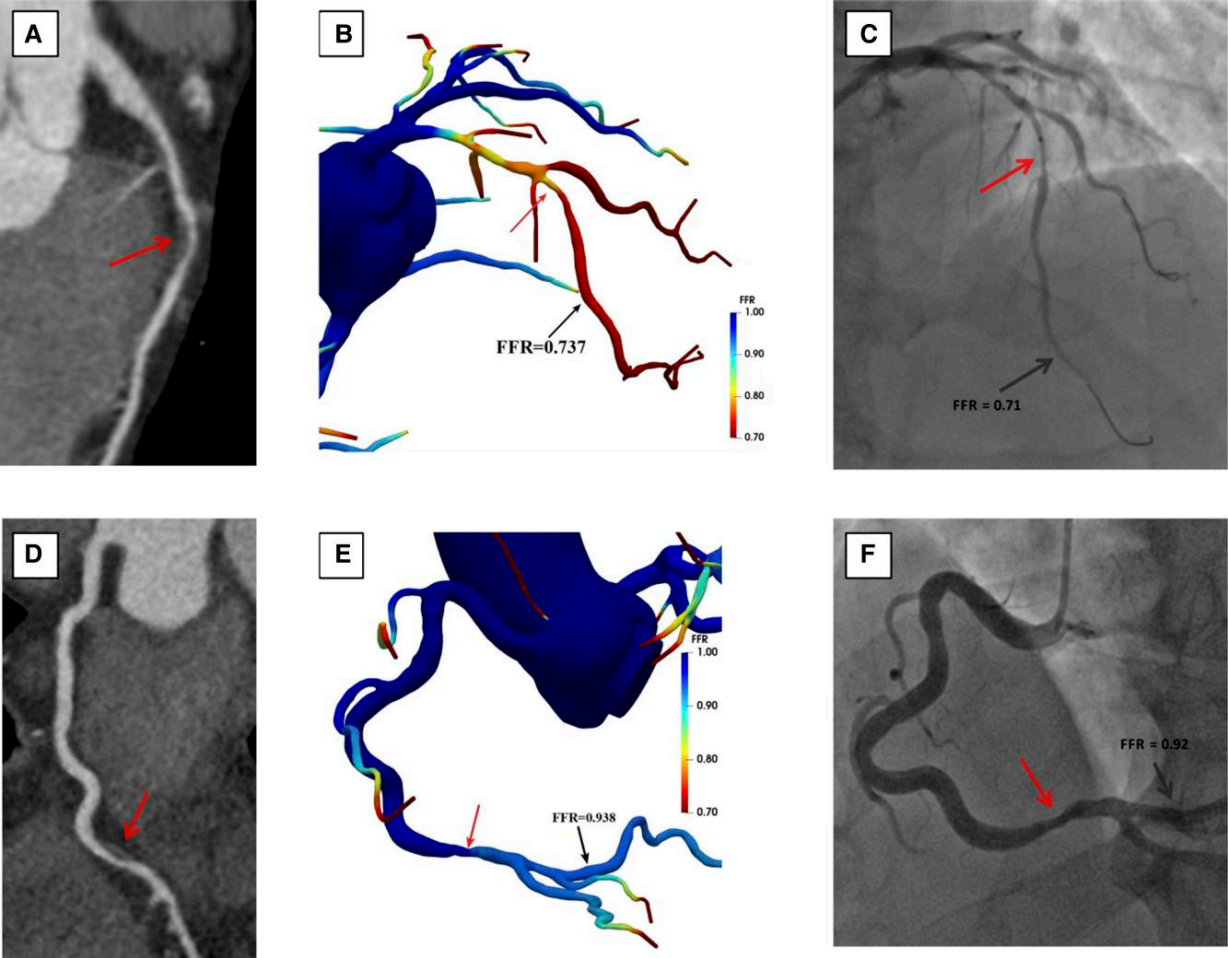
Case example. Two patients both with anatomically obstructive stenosis (arrow) on coronary computed tomography angiography (CCTA) (*A* and *D*). In the first patient (*A–C*), computation of fractional flow reserve from coronary computed tomography angiography (CT-FFR) demonstrates lesion-specific ischaemia of the middle left anterior descending (LAD) stenosis, with a value of 0.74 (*B*). Invasive coronary angiography confirms the functionally obstructive stenosis with an invasive FFR of 0.71 (*C*). In the second patient (*D–F*), CT-FFR predicts no ischaemia of the stenosis in the distal right coronary artery (RCA) with a CT-FFR value of 0.94 (*E*). Invasive FFR of 0.92 similarly demonstrates no ischaemia of RCA.

On a per-vessel basis, the diagnostic accuracy, sensitivity, specificity, PPV, and NPV of CT-FFR vs. CCTA to detect haemodynamically significant stenosis defined as FFR ≤ 0.8 are displayed in *Table 3*. Representative examples of anatomically obstructive stenosis with and without ischaemia-producing stenoses are shown in *Figure 4*. The AUC to predict FFR ≤ 0.8 on per-vessel basis of CT-FFR (0.94, 95% CI: 0.91–0.97) is significantly higher than CCTA > 50% (0.76, 95% CI: 0.71–0.82) (*P* < 0.001) (*Graphical Abstract*). Per-patient diagnostic performance of CT-FFR vs. CCTA can be seen in *Table 3*. Similar to per-vessel analyses, a higher AUC for CT-FFR was observed (0.94, 95% CI: 0.91–0.96) as compared with CCTA for per-patient discrimination (0.73, 95% CI: 0.67–0.78) (*P* < 0.001) (*Graphical Abstract*).

**Table 3:** Per-patient and per-vessel diagnostic performance of CT-FFR and CCTA

Measure	Per-vessel			Per-patient		
	CT-FFR ≤ 0.8 (95% CI)	CCTA stenosis ≥ 50% (95% CI)	*P* value	CT-FFR ≤ 0.8 (95% CI)	CCTA stenosis ≥ 50% (95% CI)	*P* value
Accuracy	89.8 (86.9–92.7)	68.4 (63.9–72.8)	<0.0001	89.9 (86.7–93.2)	71.1 (66.2–75.8)	<0.0001
Sensitivity	89.3 (83.9–94.8)	82.8 (76.1–80.1)	0.2	90.5 (84.9–96.1)	90.5 (84.9–96.1)	0.81
Specificity	90.0 (86.5–93.4)	62.3 (56.7–67.9)	<0.0001	89.7 (85.8–83.6)	62.2 (56.0–68.5)	<0.0001
PPV	79.0 (72.2–85.8)	48.1 (41.-54.9)	<0.0001	79.8 (72.6–87.0)	51.9 (44.7–59.2)	<0.0001
NPV	95.2 (92.7–97.8)	89.6 (85.3–93.8)	0.18	95.4 (92.7–98.2)	93.6 (89.7–97.4)	0.43

Values are proportion in % (95% confidence interval). FFR ≤ 0.80 was diagnostic of lesion-specific ischaemia. *N* = 338 patients and 411 for vessels.

NPV, negative predictive value; PPV, positive predictive value; other abbreviations as in *Tables 2*.

### Per-vessel diagnostic performance of CT-FFR in ‘grey zone’

Among the 133 vessels with FFR range of ‘grey zone’ (FFR 0.75–0.85), 44% (*n* = 59) exhibited ischaemia FFR. For these ‘grey zone’ lesions, CT-FFR demonstrated a diagnostic accuracy, sensitivity, specificity, PPV, and NPV of 79.7%, 81.4%, 78.4%, 75.0%, and 84.1%, respectively (*Table 4*). The AUC of CT-FFR in ‘grey zone’ analysis was 0.81 (95% CI: 0.74–0.89). CT-FFR correctly reclassified 71% (27/38) of vessels with CCTA false-positive findings as true negative findings.

**Table 4:** Diagnostic performance of CT-FFR in lesions within the grey zone of invasive FFR

	CT-FFR ≤ 0.8 (95% CI)	CCTA stenosis ≥ 50% (95% CI)	*P* value
Accuracy	79.3 (71.4–85.8)	59.3 (50.5–67.8)	0.001
Sensitivity	81.4 (69.1–90.3)	83.1 (71.0–91.6)	0.81
Specificity	78.4 (67.3–87.1)	48.7 (36.9–60.6)	0.0002
PPV	62.9 (51.9–72.7)	42.2 (36.2–48.3)	0.014
NPV	90.3 (84.4–94.2)	86.4 (77.6–92.1)	0.292

Values are proportion in % (95% confidence interval). The grey zone of invasive FFR represents values between 0.75 and 0.85. *N* = 133 for vessels. Abbreviations as in *Tables 2* and *3*.

### Parallel scalability test of the NKS method for the CT-FFR calculation

**Figure 5:**
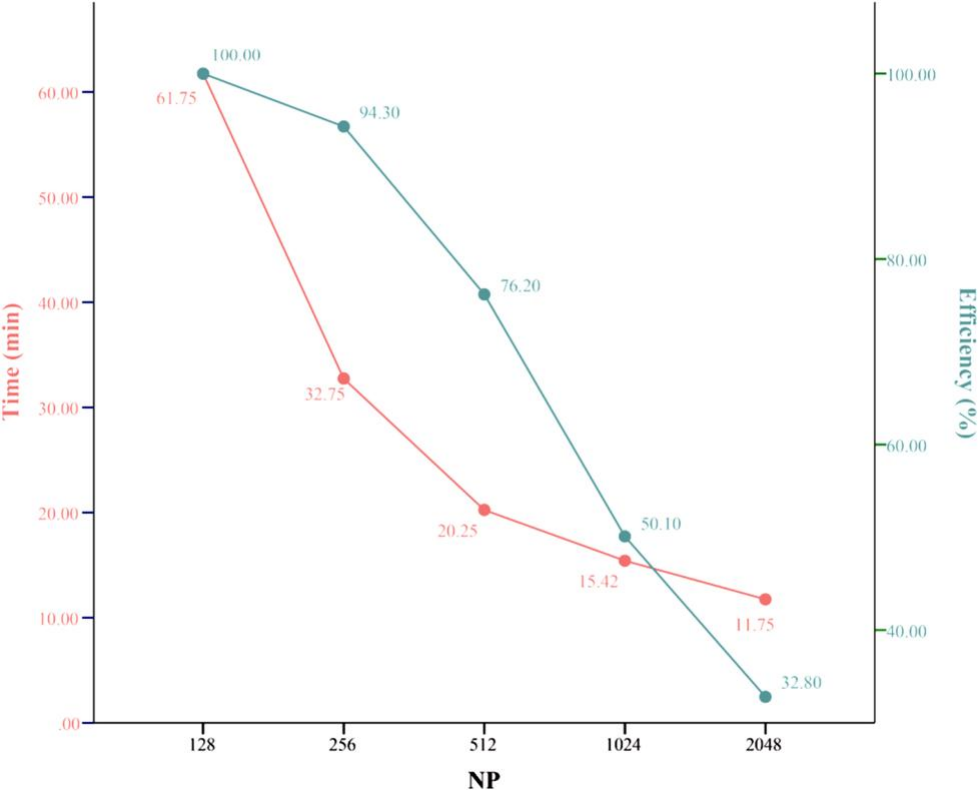
Parallel scalability of NKS for the CT-FFR calculation. In this case, NP, time, and efficiency refer to the number of processor cores, the total computational time in minutes, and the parallel efficiency, respectively. Parallel scalability of NKS for the CT-FFR calculation on a supercomputer demonstrates its effectiveness in reducing computational time indicating that the NKS method significantly reduced the calculation time, decreasing from 61.7 min with 128 processors to 11.7 min with 2048 processors when extending the reconstructed coronary tree to smaller-diameter lumen. NKS, Newton–Krylov–Schwarz; NP, number of processor cores; other abbreviations as in *Figure 1*.

**Figure 6:**
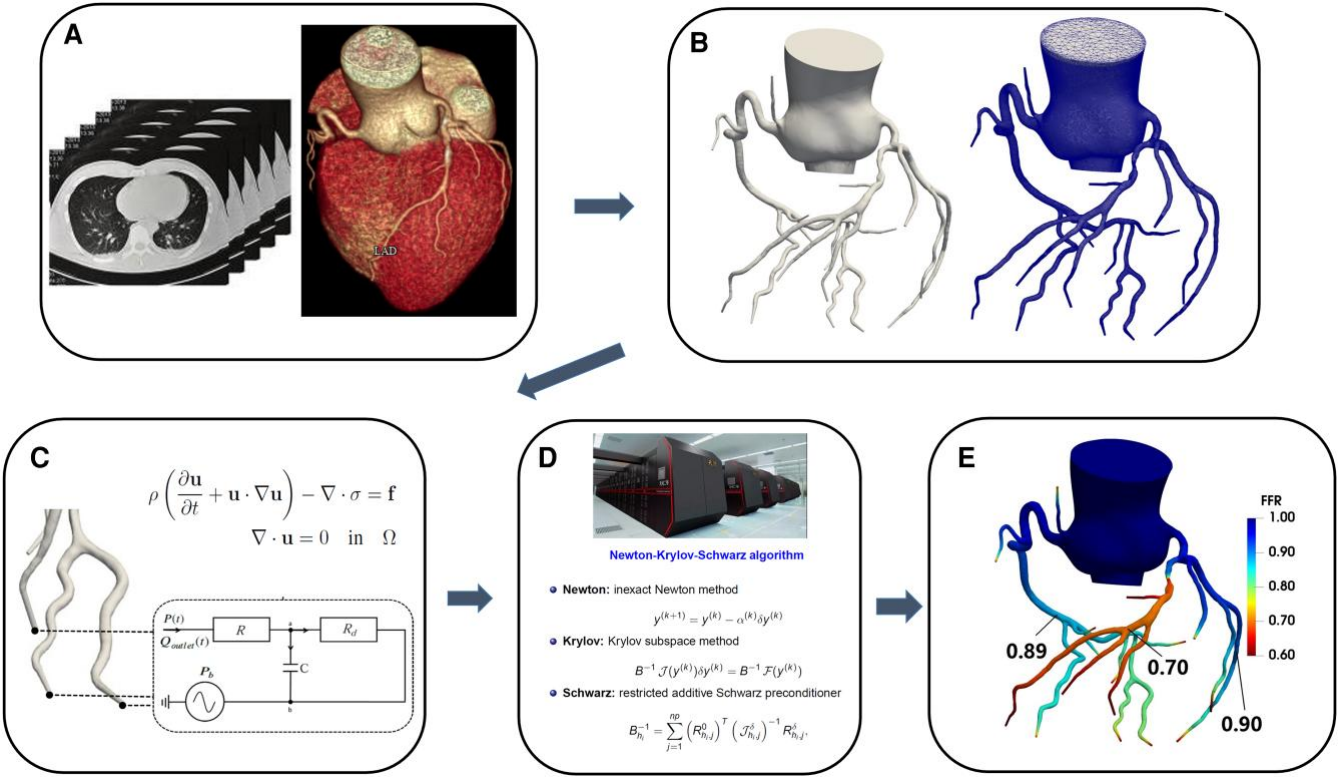
Schematic presentation of the CT-FFR methodology. (*A*) Routine coronary computed tomography angiography data are received. (*B*) Reconstruct the 3D coronary arteries and ascending aorta from CT images and generate the computational mesh in the 3D arteries. (*C*) Set boundary conditions to inlet, outlet, and wall of the computational domain. (*D*) Solve the Navier–Stokes equations for blood flow simulation on a parallel computer to calculate the FFR values. (*E*) Present the FFR values and their distribution at various locations within the coronary arteries in a clear and intuitive manner.

CT-FFR with the inclusion of smaller lumen and subject to an acceptable computation time can be calculated with the NKS method. *Figure 5* shows the parallel scalability of NKS for the CT-FFR calculation on a supercomputer, demonstrating its effectiveness in reducing computational time. The results indicate that the NKS method significantly reduced the calculation time, decreasing from 61.7 min with 128 processors to 11.7 min with 2048 processors when extending the reconstructed coronary tree to smaller-diameter lumen. This demonstrates the efficiency and scalability of the NKS method in CT-FFR calculations, as illustrated in *Figure 6*, which presents a schematic of the CT-FFR methodology.

## Discussion

This comprehensive study provides compelling evidence for the feasibility and effectiveness of a novel CT-FFR algorithm based on CFD modelling, which extends the coronary tree to smaller lumen diameters. Remarkably, this novel CT-FFR modelling exhibited a high diagnostic performance compared with invasively measured FFR in patients with intermediate stenosis. This study supports the clinical application of CT-FFR with CFD in identifying haemodynamically significant stenosis and predicting the need for subsequent revascularization. Moreover, by employing the NKS method, the computational time required for CFD calculations with smaller lumen diameters can be efficiently controlled to ∼10 min, making it clinically feasible.

A key distinction in the methodology of this CT-FFR model is the extension of the minimum diameter of the coronary lumen to 0.8 mm, whereas the minimum lumen diameter of the reconstructed arteries was 1.2–1.5 mm in previous reported CFD models.^7,8,17,19–21^ In CFD, the effects of blood flow in the downstream arterioles and capillaries are coupled through outlet boundary conditions. Incorrect outlet boundary conditions can introduce significant uncertainty in simulations of coronary blood flow.^22^ Therefore, extending the reconstructed coronary tree to smaller-diameter lumen provides a more realistic model and improves the accuracy of CT-FFR estimation. Another notable aspect was the utilization of the NKS method, which effectively reduced the computational time from 61.7 min with 128 processors to 11.7 min with 2048 processors for the CT-FFR calculation in this study. In comparison, HeartFlow CT-FFR required 1–4 h per examination depending on disease burden and CT image quality.^17^ Approximately 1 h was needed for workstation-based CFD model,^23^ while one-dimensional CFD-based models took around 30 min.^8^ Theoretically, increasing the availability of computing resources can further reduce the total time, although with limiting efficiency. Thus, the appropriate number of processors can be selected based on medical requirements and available computational resources, facilitating the implementation of CT-FFR across different healthcare settings.

From previous results, the per-vessel accuracy of CFD-based CT-FFR ranged from 71–86%.^8,9,17,19–21,23^ For ML-based CT-FFR, a comparable accuracy of 78–83% has been exhibited on a per-vessel basis for the detection of haemodynamically significant stenosis.^9,24^ In the present study, a new method by extending the reconstructed coronary tree to a smaller lumen diameter improved 3D reconstruction and blood flow calculation, and the per-vessel diagnostic accuracy in the present study (89.3%) was a mildly higher than the reported performance of both CFD-based or ML-based CT-FFR in determining the haemodynamic significance of a coronary stenosis.

In our study, a subgroup of 133 stenoses (32.4%) fell within the ‘grey zone’ of invasive FFR, with values ranging between 0.75 and 0.85. Crucially, the agreement between CT-FFR and FFR depends on the stenosis characteristics.^25^ Furthermore, the more intermediate the values, the lower the agreement, even for repeatability of invasive FFR itself.^26^ Our analysis revealed that CT-FFR demonstrated lower per-vessel accuracy (79.7%) within the grey zone. However, it still outperformed CCTA alone in this regard. Additionally, this prototype of CT-FFR also exhibits higher diagnostic accuracy (79.7% vs. 67.6%) within the grey zone FFR compared with the on-site CFD-based CTA-FFR.^27^ This improvement can be attributed to the extension of the reconstructed coronary tree to smaller lumen diameters, which increases the computational domain and reduces the impact of erroneous boundary conditions on CT-FFR calculations.

Our findings provide strong evidence that patients who test negative for myocardial ischaemia using CT-FFR have a low likelihood of having the condition, as indicated by a high NPV of 95%. Notably, our study also demonstrated excellent reproducibility, with little variability observed between different observers and on repeated measures. The excellent diagnostic performance of the present findings highlights the promising potential of CT-FFR in determining the haemodynamic significance of CAD in a wide variety of clinical settings, supporting the potential role of CCTA with CT-FFR as a reliable gatekeeper to ICA and revascularization.

### Study limitations

In the present study, not all vessels in the enrolled patients underwent assessment. The vessels with diameter stenosis < 30% or >90% were excluded from physiological evaluations as they were deemed unnecessary. The number of eligible patients who were not recruited, but met the criteria for study inclusion (such as undergoing ICA after coronary CTA), was not documented, which introduces the possibility of site-level selection bias based on CCTA findings. However, considering the pre-test risk of CAD, as well as the stenosis spectrum and prevalence of disease in this trial, the findings can still be considered applicable to a broader population. Patients with acute coronary syndromes and previous coronary intervention or bypass surgery were excluded from the present study. Thus, generalizability of CT-FFR to these specific populations of patients with CAD is unknown. It is important to note that this study did not analyse the costs or cost-effectiveness of CT-FFR. Additionally, there is limited knowledge regarding the comparative performance of CT-FFR against other functional imaging modalities. Previous studies have shown that HeartFlow FFR_CT_ has demonstrated superior diagnostic performance than other non-invasive traditional functional imaging.^28^ Therefore, to establish the diagnostic accuracy of CT-FFR more comprehensively, it is necessary to conduct direct head-to-head comparisons between CT-FFR and other functional imaging modalities in future research.

## Conclusion

This prospective multicentre study demonstrates that the novel CFD-based CT-FFR approach by extending the reconstructed coronary tree to a smaller lumen diameter has excellent diagnostic performance in accurately identifying the lesion-specific ischaemia, as evidenced by its high sensitivity and specificity. Additionally, with the application of NKS method for the CT-FFR calculation, the computational time can be optimized to ∼10 min, making it clinically feasible and efficient.

## Competency in medical knowledge

Among patients with intermediate coronary stenosis, the novel CT-FFR model with the inclusion of smaller lumen provides excellent diagnostic accuracy in detecting haemodynamically significant CAD. The integration of the NKS method ensures that the computation time remains within an acceptable range for potential clinical applications in the future.

## Translational outlook

Further clinical trials are needed to validate the prognostic value of this novel. CT-FFR in patients with CAD and to compare the performance of potential outcomes of patients with other non-invasive functionally assessment techniques.

## Supplementary data

Supplementary data are available at *European Heart Journal – Imaging Methods and Practice* online.

## Supplementary Material

qyae106_Supplementary_Data

## Data Availability

The data underlying this article can be shared upon reasonable request from the corresponding author.

## Lead author biography

Dr Zeng Mengsu is a prominent radiologist with extensive achievements in radiology. He has published over 300 academic papers, including more than 100 indexed in SCI, and has authored 20 medical monographs. Dr Zeng holds significant roles in various medical societies, including committee member of the Radiology Branch of the Chinese Medical Association and Deputy Head of the Abdominal Imaging Group. He serves as Vice Editor-in-Chief of the Journal of Clinical Radiology and is on the editorial boards of several prestigious journals, including the Chinese Journal of Radiology.
