# Supplementary material for: CT-FFR by expanding coronary tree with Newton–Krylov–Schwarz method to solve the governing equations of CFD
Source: Eur Heart J Imaging Methods Pract. 2024 Oct 24;2(3):qyae106. doi: 10.1093/ehjimp/qyae106 (PMC11547952; doi:10.1093/ehjimp/qyae106)
Supplement: qyae106_Supplementary_Data [file qyae106_supplementary_data.docx]

**SUPPLEMENTARY MATERIAL**

**CT-FFR Analysis**

The CT-FFR calculations begin with the reconstruction of the 3D coronary artery based on CCTA images, including all arteries with a lumen diameter larger than 0.8mm, using the commercial software Materialise Mimics. Subsequently, an unstructured tetrahedral mesh was generated by the commercial software Ansys ICEM CFD to discretize the 3D artery, which consists of millions of elements. The blood flow is assumed to follow Newtonian behavior, characterized by constant viscosity, and is described by the time-dependent incompressible Navier-Stokes equations with specific boundary conditions. A no-slip condition, imposing zero velocity, is enforced at the vessel walls. The inlet boundary condition is defined by a patient-specific, time-dependent inflow rate derived from patient-specific heart rate and left ventricular myocardium volume. At the outlet boundaries, the three-element Windkessel model is employed, and its parameters are determined based on the left ventricular myocardium volume. To solve the time-dependent incompressible Navier-Stokes equations in the CFD simulation, a first-order finite element method was employed for the spatial discretization and a second-order fully implicit Backward Differentiation Formula (BDF) scheme method was utilized for the temporal discretization. At each time step, a large finite-dimensional nonlinear system emerged, which was solved using the highly parallel scalable and robust NKS method which comprise three key components (1). Firstly, an inexact Newton method, supplemented by a line search method, manages the nonlinear system. Secondly, to handle the non-symmetric Jacobian system arising within each Newton iteration, a Krylov subspace method is introduced; specifically, GMRES method is used. Lastly, to enhance the convergence speed, an overlapping Schwarz method acts as a preconditioner, accelerating the Krylov method's performance. The simulation algorithms are implemented using the programming languages C and MPI. Further elaboration on the algorithmic details can be found in our previous publication (2).

CT-FFR is calculated by the ratio of Pd (pressure at the distal end of the stenosis in the coronary artery) and Pa (pressure at the entrance of the coronary artery) measured from the results of the CFD simulation. The CT-FFR values are calculated based on the derived pressures along the length of the vessel, considering a minimum diameter of 0.8mm. This process generates a color-coded 3D model, enabling the determination of CT-FFR values at any desired location within the coronary artery. To ensure accurate correlation between the lesion's location on CCTA images and the 3D CT-FFR coronary meshes, an 18-segment coronary artery model was employed (3). This model facilitated the precise co-registration of the coronary lesion between the CCTA images and the 3D CT-FFR model. Functionally significant CAD was defined as CT-FFR ≤0.80.

**Reference**

1. Wu X, Wu B, He W, et al. Expanding the coronary tree reconstruction to smaller arteries improves the accuracy of FFRCT. Eur Radiol. 2021;31(12):8967-8974.
2. Chen R, Wu B, Cheng Z, et al. A parallel non-nested two-level domain decomposition method for simulating blood flows in cerebral artery of stroke patient. Int J Numer Method Biomed Eng. 2020;30:e3392.
3. Leipsic J, Abbara S, Achenbach S, et al. SCCT guidelines for the interpretation and reporting of coronary CT angiography: a report of the Society of Cardiovascular Computed Tomography Guidelines Committee. J Cardiovasc Comput Tomogr 2014;8:342–58.
